# Switching from complement inhibitors in AQP4-IgG–positive NMOSD: clinical characteristics, an operational “cluster phase” and potential strategies to prevent therapeutic gaps

**DOI:** 10.1186/s12883-026-05130-x

**Published:** 2026-06-29

**Authors:** Kenzo Sakurai, Kei Kaburagi, Kenji Isahaya, Hisanao Akiyama, Yoshihisa Yamano

**Affiliations:** Department of Neurology, St. Marianna University School of Medicine, 2-16-1, Sugao, Miyamae, Kawasaki, Kanagawa 216-8511 Japan

**Keywords:** Neuromyelitis optica spectrum disorder, AQP4-IgG, Complement inhibitor, Cluster phase, Switching, Relapse, Bridging strategy, No-gap strategy

## Abstract

**Background:**

Neuromyelitis optica spectrum disorder (NMOSD) can cause severe neurological disability after a single relapse. Although relapse rates have markedly decreased with the emergence of targeted biologics, treatment switches are increasingly encountered due to long-term treatment considerations and adverse events. However, safe switching strategies, especially within 12 months after a relapse, which we defined operationally as the “cluster phase”, have not been established. We investigated relapse risk and potential preventive strategies when switching from complement inhibitors in AQP4-IgG–positive NMOSD.

**Methods:**

We retrospectively reviewed patients with AQP4-IgG–positive NMOSD treated at St. Marianna University School of Medicine who underwent at least one biologic switch. Clinical records were assessed for patient characteristics, treatment history, timing of switching, and relapse occurrence. Particular attention was given to switching from complement inhibitors during the cluster phase.

**Results:**

Among 14 patients who switched biologics, 5 switched from complement inhibitors, 4 switched between complement inhibitors, 2 between B-cell–depleting therapies, and 3 from IL-6 receptor inhibitors. Six of the 14 switches occurred during the cluster phase, including 3 from complement inhibitors (all to B-cell–depleting agents). One of the two patients switched without bridging therapy during the cluster phase experienced optic neuritis relapse, while no relapses occurred in the other 13 switching cases. This observation suggests a possible vulnerability during the cluster phase, although definitive conclusions cannot be drawn due to the small sample size.

**Conclusions:**

Switching from complement inhibitors during the cluster phase may be associated with an increased susceptibility to relapse. Bridging strategies, such as plasma exchange or temporary biologic overlap, may help reduce the likelihood of therapeutic gaps, but these findings remain hypothesis-generating. Clinicians should proactively discuss future switching scenarios and develop long-term treatment plans with patients.

**Supplementary Information:**

The online version contains supplementary material available at https://doi.org/10.1186/s12883-026-05130-x.

## Introduction

Neuromyelitis optica spectrum disorder (NMOSD) is an autoimmune inflammatory disease of the central nervous system characterized by pathogenic immunoglobulin G antibodies against aquaporin-4 (AQP4). Patients are at risk of severe neurological disability from even a single relapse [1], therefore achieving zero relapse is a critical therapeutic goal. The introduction of targeted biologics in recent years has substantially reduced relapse rates in AQP4-IgG–positive NMOSD.

Complement inhibitors such as eculizumab and ravulizumab exert rapid clinical efficacy and are often selected in the acute disease stage to achieve prompt disease control [2]. In contrast, interleukin-6 (IL-6) receptor inhibitors, such as satralizumab, and B-cell–depleting agents, including inebilizumab and rituximab, are thought to suppress upstream immune mechanisms and may offer broader immunomodulatory effects [3, 4]; therefore, switching from complement inhibitors to these agents is increasingly encountered in clinical practice once acute disease activity has stabilized.

However, complement inhibition acts on the downstream effector phase of the disease without suppressing the production of AQP4-IgG. Consequently, switching away from complement inhibitors may theoretically increase relapse risk, particularly when circulating pathogenic antibodies remain active. Evidence regarding optimal switching strategies from complement inhibitors in NMOSD remains limited, and there is no consensus on best practices. Of particular clinical importance is the “cluster phase”, which we defined operationally as the first 12 months after a relapse, based on prior observations that relapse activity tends to cluster within this period [5]. This concept has not been universally adopted, and therefore its use in this study should be interpreted as a pragmatic framework for assessing relapse vulnerability rather than an established definition.

In this study, we examined our real-world experience with switching from complement inhibitors to other biologics in patients with AQP4-IgG–positive NMOSD, with a specific focus on outcomes during the cluster phase.

## Methods

### Study design and patients

We conducted a single-center retrospective observational study of patients with AQP4-IgG–positive NMOSD treated at the Department of Neurology, St. Marianna University School of Medicine. Among patients who had been treated with biologics up to October 2025, those who experienced at least one switch between biologic therapies were included. Patients who underwent more than one switch were evaluated only at the time of their first switch. AQP4-IgG–seronegative patients were excluded. The diagnosis of NMOSD was based on the 2015 International Panel for NMO Diagnosis criteria. We analyzed only the first switch in each patient because repeated switching events within the same patient are not statistically independent, and because the cluster phase is defined relative to the most recent relapse; restricting the analysis to the index switch therefore minimized within-patient confounding and provided the most consistent assessment of relapse risk at the point of transition.

### Treatments and definitions

Biologic agents approved for NMOSD in Japan were included:

- complement inhibitors: eculizumab, ravulizumab
- IL-6 receptor inhibitor: satralizumab
- B-cell–depleting agents: inebilizumab, rituximab

Data regarding the first biologic switch were collected, including the initial agent, switch destination, timing of switch, and concurrent therapies.

The cluster phase was defined as within 12 months after the last relapse, in reference to prior reports suggesting that NMOSD relapses often cluster early after an attack. This definition is not an internationally standardized criterion but used as a clinically meaningful time window for exploratory analysis.

Switching events were classified as occurring during the cluster phase or outside this period (non-cluster phase).

### Data collection

Medical records were reviewed to extract demographic and clinical information, including age, sex, Expanded Disability Status Scale (EDSS) score [6], disease duration, number of previous relapses, and coexisting autoimmune diseases. Treatment-related data included:

- biologics used before and after switching
- interval between last relapse and switching
- oral corticosteroid dose at switching
- concomitant use of immunosuppressants
- bridging therapies, including plasma exchange or short-term biologic overlap, when applied

Serum AQP4-IgG antibody titers measured at the time of diagnosis (by enzyme-linked immunosorbent assay) were also recorded.

Relapse was defined as new or worsening neurological symptoms lasting > 24 h without an alternative explanation, accompanied by MRI or clinical confirmation.

### Ethics

This study was approved by the Institutional Review Board of St. Marianna University School of Medicine (Approval No. 6092). Written informed consent was obtained from all patients. This study was reported in accordance with the STROBE guidelines (see Additional file 1).

## Results

**Fig. 1:**
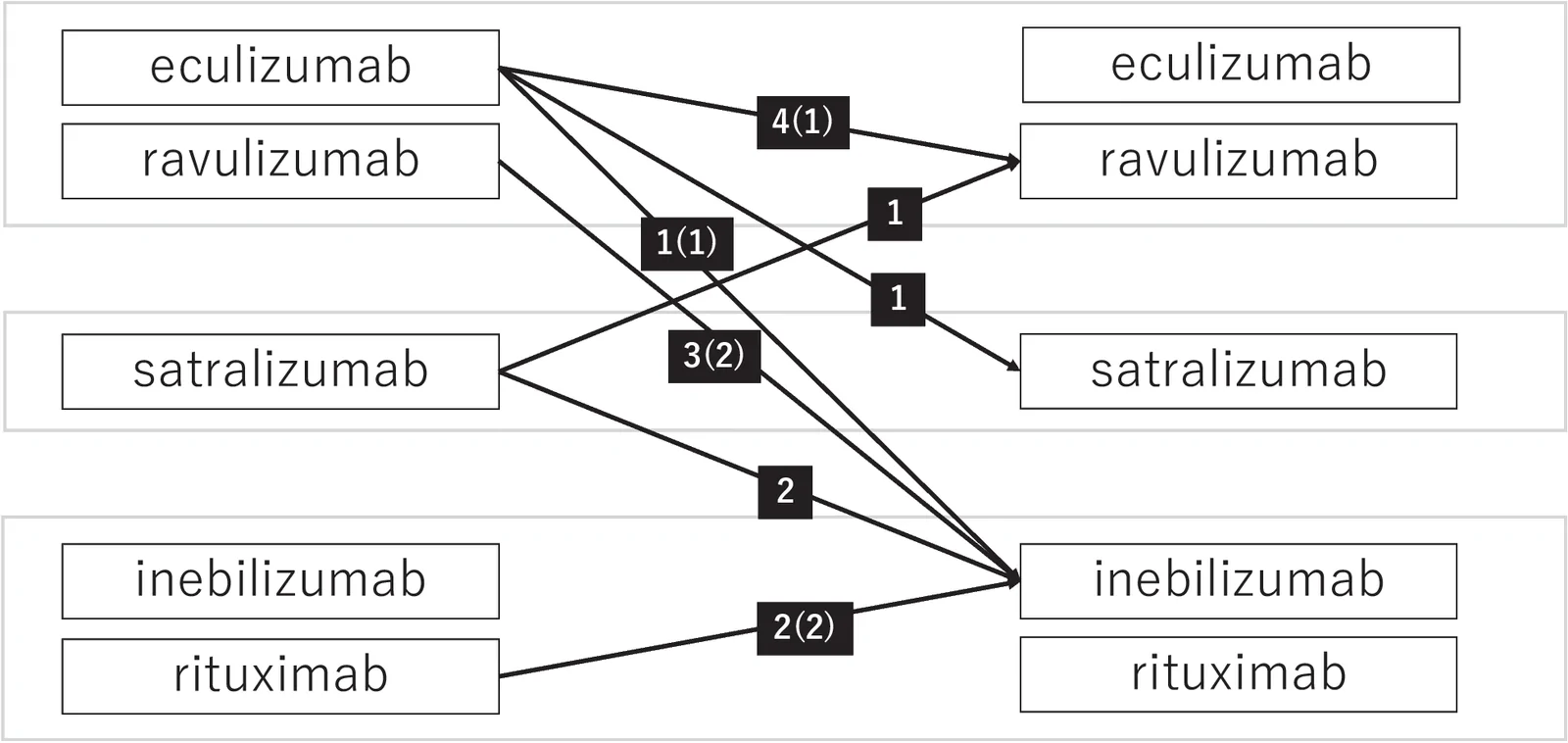
Switching pathways from complement inhibitors. Arrows indicate switching directions. Numbers in parentheses represent switches occurring during the operationally defined cluster phase (within 12 months after the last relapse). Abbreviations: ECU, eculizumab; RAV, ravulizumab; SAT, satralizumab; INE, inebilizumab; RTX, rituximab

**Fig. 2:**
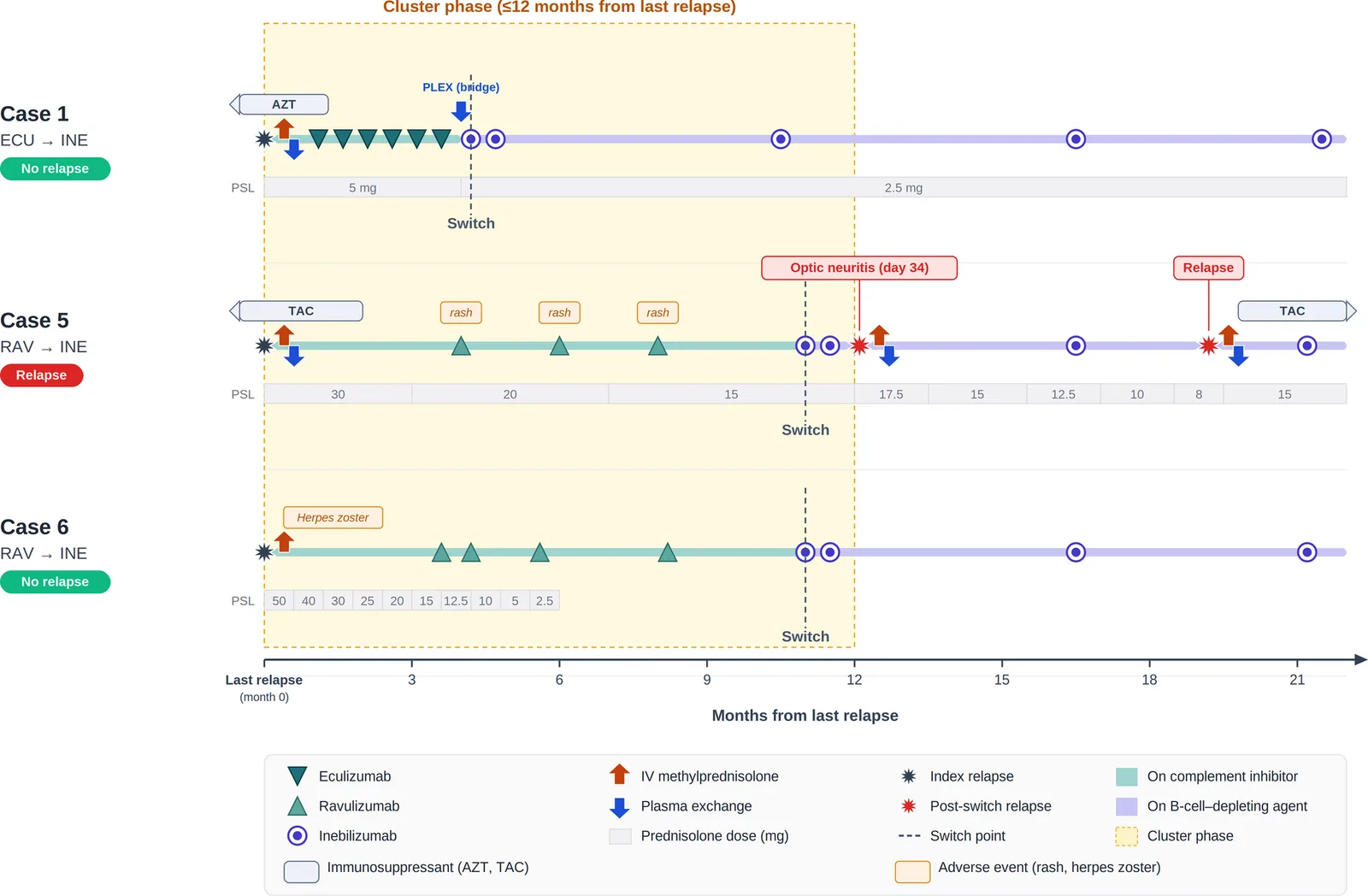
Clinical timeline of cluster-phase switching cases. The timing of biologic transition and relapse events are shown. Abbreviations: ECU, eculizumab; RAV, ravulizumab; INE, inebilizumab; PLEX, plasma exchange; ON, optic neuritis. The shaded band denotes the cluster phase (within 12 months after the last relapse). For each case, the maintenance biologic, switch point, index and post-switch relapses, acute treatments, oral prednisolone dose, immunosuppressant use, and adverse events are shown. Additional abbreviations: IVMP, intravenous methylprednisolone; AZT, azathioprine; TAC, tacrolimus

A total of 38 patients had received biologic therapies, of whom 3 discontinued treatment and 21 did not switch biologics during follow-up. Fourteen patients (all female; mean age 53.1 ± 16.5 years) underwent at least one biologic switch and were included in this analysis. Median EDSS score was 5.0 (IQR 0–7.5), mean disease duration was 7.4 ± 8.4 years, median number of prior relapses was 1 (IQR 1–10), and 4 patients (28.6%) had coexisting autoimmune diseases. The first biologic used was eculizumab in 6 patients, ravulizumab and satralizumab in 3 patients each, rituximab in 2 patients, and none received inebilizumab as first-line therapy (Table 1). Among the 14 switching events, 5 were from complement inhibitors to other agents, 4 between complement inhibitors, 2 between B-cell–depleting agents, and 3 from an IL-6 receptor inhibitor to either a B-cell–depleting therapy (*n* = 2) or a complement inhibitor (*n* = 1). Among the 5 patients who switched from complement inhibitors, 3 switched during the cluster phase and 2 during the non-cluster phase. All 3 of these cluster-phase switches from complement inhibitors were to B-cell–depleting therapies (Fig. 1). At the time of switching, the median oral corticosteroid dose was 3.75 mg (IQR 0–30), and 2 patients (14.2%) were receiving additional immunosuppressants. Among the 3 cluster-phase switches from complement inhibitors, one patient underwent plasma exchange and subsequently started inebilizumab the next day without relapse, while another patient switched without bridging and developed optic neuritis 34 days later (Fig. 2). These observations may indicate a potential vulnerability during the cluster phase, although the limited sample size precludes firm conclusions.

**Table 1:**
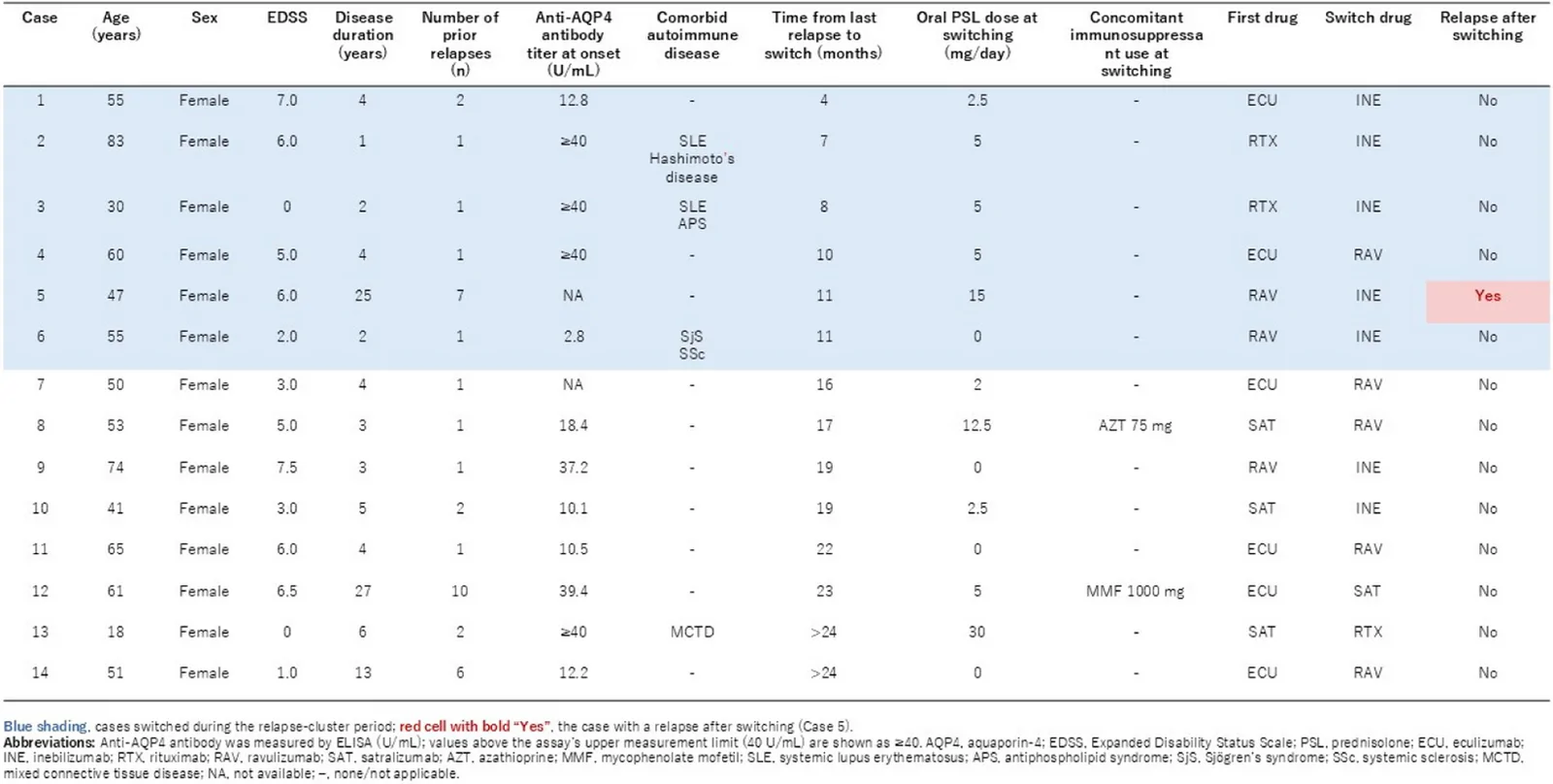
Patient characteristics at the time of biologic switching

Overall, 6 of the 14 switches occurred during the cluster phase—3 from complement inhibitors, 1 between complement inhibitors, and 2 between B-cell–depleting agents—whereas the remaining 8 switches, including all 3 from IL-6 receptor inhibitors, occurred during the non-cluster phase. The phenotype and severity of the last attack before switching in these 6 cluster-phase cases are summarized in Table 2: five presented as myelitis and one as optic neuritis, with severity graded from mild to severe, and all required intravenous methylprednisolone, with plasma exchange added in five and intravenous immunoglobulin in three.

**Table 2:**
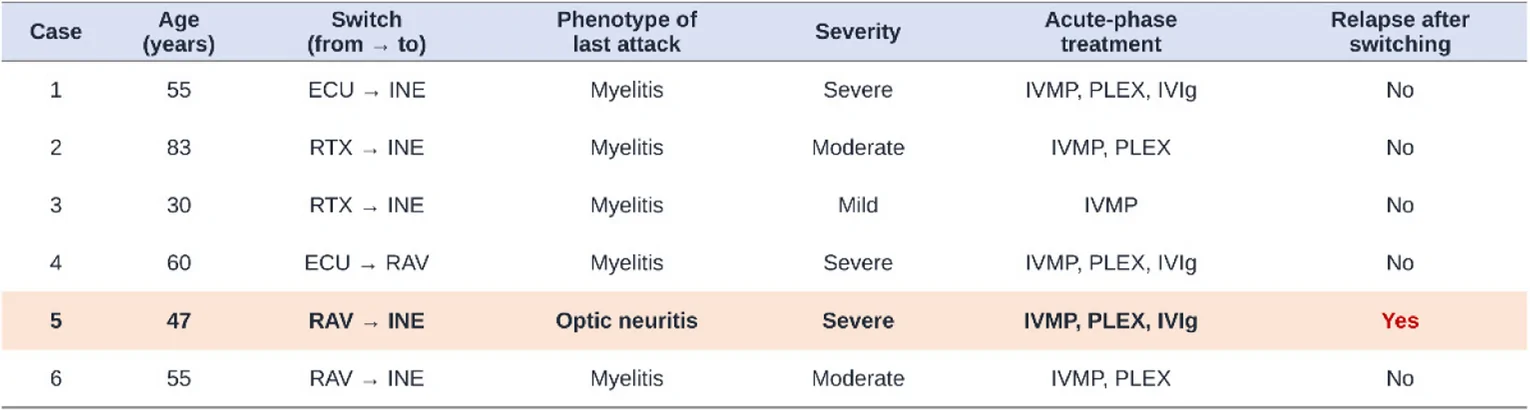
Clinical features of the last attack before switching in the six cluster-phase cases

The single patient who relapsed (Case 5) was a 47-year-old woman with the longest disease duration (25 years) and the highest number of prior relapses (7) in the cohort. Her last attack before switching was optic neuritis, and she switched from ravulizumab to inebilizumab without bridging therapy 11 months after that attack, while receiving oral prednisolone 15 mg/day; she had no coexisting autoimmune disease and was not receiving additional immunosuppressants. She developed optic neuritis again 34 days after switching. Among the 4 patients with a coexisting autoimmune disease, treatment was directed at NMOSD in 3, whereas only Case 13 received high-dose corticosteroid (prednisolone 30 mg/day) for mixed connective tissue disease. Diagnostic AQP4-IgG titers for all patients are shown in Table 1; titers were not re-measured at the time of relapse.

## Discussion

In this retrospective study, we observed that switching from complement inhibitors during the cluster phase may be associated with increased relapse susceptibility in AQP4-IgG-positive NMOSD. Although inebilizumab is effective in reducing CD19-positive B cells, its full therapeutic effect may take 1–2 months to establish, during which early relapses have also been reported in the N-MOmentum trial [3]. A similar trend has been reported with satralizumab, suggesting that a therapeutic gap may arise when transitioning biologics with slower onset of action [4]. Therefore, establishing agent-specific switching strategies is critical to maintaining relapse-free disease control. However, this finding is based on a single relapse event, and therefore should be interpreted with caution and viewed as hypothesis-generating rather than confirmatory.

Complement inhibitors such as eculizumab and ravulizumab suppress complement-mediated astrocyte injury, acting at the downstream effector phase of NMOSD pathophysiology without reducing AQP4-IgG production [2]. Consequently, when complement inhibition is withdrawn, residual or newly produced AQP4-IgG may drive complement activation again, theoretically increasing relapse risk. Indeed, prior reports have described early relapses after discontinuation of complement inhibitors [7], and a severe relapse has been documented after switching from eculizumab to satralizumab [8]. In contrast, switching among B-cell–depleting therapies or from B-cell–depleting agents to satralizumab has generally been reported as safe [9, 10], highlighting the unique vulnerability associated with withdrawal of complement inhibition. Nevertheless, our data do not allow causal inference, and additional studies are needed.

The risk of relapse is highest within the first year after an attack, referred to as the cluster phase [5], and careful treatment strategies are warranted during this period. Consistent with prior knowledge, the only relapse observed in our cohort occurred during a cluster-phase switch. In contrast, switching outside the cluster phase appeared to be safer, likely reflecting reduced disease activity and lower AQP4-IgG levels in remission [11, 12]. Nonetheless, late relapses can still occur after prolonged remission [13], underscoring the importance of avoiding therapeutic gaps even outside the cluster period. Notably, the relapsing patient differed from the other five cluster-phase cases in several respects: she had the longest disease duration and the highest number of prior relapses, presented with optic neuritis (the only such case, the others presenting with myelitis), and switched without bridging therapy—features that may reflect higher intrinsic disease activity and greater susceptibility to a therapeutic gap during transition.

Although optimal switching strategies after complement inhibitors have not been established, our findings suggest that bridging approaches may be beneficial, particularly during the cluster phase. Plasma exchange, which rapidly reduces circulating AQP4-IgG, prevented relapse in one of our cluster-phase switch cases. In addition, theoretical strategies include temporary biologic overlap or the future use of neonatal Fc receptor inhibitors, which rapidly lower pathogenic IgG levels [14]. Recent case reports also support short-term rituximab overlap preceding withdrawal of complement inhibitors [15]. Importantly, washout periods should be avoided when transitioning off complement inhibitors. Escalation of oral corticosteroids, immunosuppressants, or intravenous methylprednisolone may provide partial benefit but may not be sufficient for relapse prevention in NMOSD, where treatment gaps are unacceptable.

Our study further emphasizes that planning for future treatment transitions should begin when initiating complement inhibitors. Shared decision-making with patients regarding long-term treatment goals, potential switching scenarios, and bridging strategies is essential to maintain continuous protection against relapse. Proactive transition planning may represent a new standard of care for biologic sequencing in NMOSD.

This study has limitations, including its single-center retrospective design and small sample size. Switching strategies were individualized based on clinical judgment, and serial biomarkers such as AQP4-IgG titers were not uniformly available. Overall, our findings suggest a possible trend that switching during the cluster phase requires additional caution. Because only the first switch in each patient was analyzed, information from subsequent switching events was not captured, which may limit the completeness and generalizability of our observations.

## Conclusion

Switching from complement inhibitors during the cluster phase may carry an increased risk of relapse, although the evidence is limited and based on a single event. Bridging approaches, such as plasma exchange or short-term biologic overlap, may help reduce therapeutic gaps, but should be considered exploratory rather than established practice. Switching outside the cluster phase appeared safer in this small cohort. Careful planning and shared decision-making from the initiation of complement inhibitor therapy remain essential for maintaining continuous relapse prevention.

## Supplementary Information

Additional file 1: STROBE Checklist:STROBE Statement—Checklist of items that should be included in reports of observational studies.

## Data Availability

The data supporting the findings of this study are available from the corresponding author upon reasonable request. Individual patient-level data cannot be publicly shared due to privacy restrictions.

## Abbreviations

NMOSD: Neuromyelitis optica spectrum disorder
AQP4: Aquaporin-4
AQP4-IgG: Anti-aquaporin-4 immunoglobulin G
IL-6: Interleukin-6
EDSS: Expanded Disability Status Scale
MRI: Magnetic resonance imaging
PLEX: Plasma exchange
IVMP: Intravenous methylprednisolone
PSL: Prednisolone
FcRn: Neonatal Fc receptor
