# Supplementary material for: Switching from complement inhibitors in AQP4-IgG–positive NMOSD: clinical characteristics, an operational “cluster phase” and potential strategies to prevent therapeutic gaps
Source: BMC Neurol. 2026 Jun 29;26:584. doi: 10.1186/s12883-026-05130-x (PMC13580005; doi:10.1186/s12883-026-05130-x)
Supplement: Supplementary file 1 [file 12883_2026_5130_MOESM1_ESM.docx]

**STROBE Statement — Checklist of items that should be included in reports of observational studies**

*Reference: von Elm E, Altman DG, Egger M, Pocock SJ, Gøtzsche PC, Vandenbroucke JP. The Strengthening the Reporting of Observational Studies in Epidemiology (STROBE) Statement: guidelines for reporting observational studies.*

**Manuscript:** Switching from Complement Inhibitors in AQP4-IgG–Positive NMOSD: Clinical Characteristics, an Operational “Cluster Phase,” and Potential Strategies to Prevent Therapeutic Gaps

| **Section/Topic** | **Item #** | **STROBE Recommendation** | **Reported on page/section** |
| --- | --- | --- | --- |
| **Title and abstract** | 1 | (a) Indicate the study's design with a commonly used term in the title or the abstract (b) Provide in the abstract an informative and balanced summary of what was done and what was found | Title page and Abstract (Background, Methods, Results, Conclusions) |
| **Introduction** |  |  |  |
| **Background/rationale** | 2 | Explain the scientific background and rationale for the investigation being reported | Introduction, paragraphs 1–3 |
| **Objectives** | 3 | State specific objectives, including any prespecified hypotheses | Introduction, paragraph 4 |
| **Methods** |  |  |  |
| **Study design** | 4 | Present key elements of study design early in the paper | Methods, 'Study design and patients', paragraph 1 |
| **Setting** | 5 | Describe the setting, locations, and relevant dates, including periods of recruitment, exposure, follow-up, and data collection | Methods, 'Study design and patients', paragraph 1 (single-center, St. Marianna University, up to October 2025) |
| **Participants** | 6 | (a) Give the eligibility criteria, and the sources and methods of selection of participants | Methods, 'Study design and patients', paragraphs 1–2 (AQP4-IgG-positive NMOSD, at least one biologic switch, first switch only) |
| **Variables** | 7 | Clearly define all outcomes, exposures, predictors, potential confounders, and effect modifiers. Give diagnostic criteria, if applicable | Methods, 'Treatments and definitions' and 'Data collection' (cluster phase definition, relapse definition, biologic agents, EDSS, switching timing) |
| **Data sources/measurement** | 8 | For each variable of interest, give sources of data and details of methods of assessment (measurement). Describe comparability of assessment methods if there is more than one group | Methods, 'Data collection' (medical records review, EDSS, MRI confirmation) |
| **Bias** | 9 | Describe any efforts to address potential sources of bias | Discussion, limitations paragraph (single-center, individualized switching strategies, no serial biomarkers) |
| **Study size** | 10 | Explain how the study size was arrived at | Results, paragraph 1 (38 biologic-treated patients; 14 met inclusion criteria). No formal sample size calculation (exploratory study) |
| **Quantitative variables** | 11 | Explain how quantitative variables were handled in the analyses. If applicable, describe which groupings were chosen and why | Methods, 'Treatments and definitions' (cluster phase: within 12 months; non-cluster phase: outside this period) |
| **Statistical methods** | 12 | (a) Describe all statistical methods, including those used to control for confounding (b) Describe any methods used to examine subgroups and interactions (c) Explain how missing data were addressed (d) If applicable, describe analytical methods taking account of sampling strategy (e) Describe any sensitivity analyses | Not applicable — descriptive case series with no formal statistical comparisons. Data are presented as means ± SD, medians with IQR, and frequencies |
| **Results** |  |  |  |
| **Participants** | 13 | (a) Report numbers of individuals at each stage of study — e.g., numbers potentially eligible, examined for eligibility, confirmed eligible, included in the study, completing follow-up, and analysed (b) Give reasons for non-participation at each stage (c) Consider use of a flow diagram | Results, paragraph 1 (38 biologic-treated → 3 discontinued, 21 no switch → 14 included). No flow diagram included |
| **Descriptive data** | 14 | (a) Give characteristics of study participants (e.g., demographic, clinical, social) and information on exposures and potential confounders (b) Indicate number of participants with missing data for each variable of interest | Results, paragraph 1 and Table 1 (age, sex, EDSS, disease duration, prior relapses, comorbid autoimmune diseases, PSL dose, immunosuppressants) |
| **Outcome data** | 15 | Report numbers of outcome events or summary measures | Results, paragraphs 2–3 (1/14 relapse overall; 1/3 cluster-phase switches from complement inhibitors) |
| **Main results** | 16 | (a) Give unadjusted estimates and, if applicable, confounder-adjusted estimates and their precision (e.g., 95% confidence interval). Make clear which confounders were adjusted for and why they were included (b) Report category boundaries when continuous variables were categorized (c) If relevant, consider translating estimates of relative risk into absolute risk for a meaningful time period | Results, paragraphs 2–3 and Figures 1–2 (descriptive; no adjusted estimates due to small sample size) |
| **Other analyses** | 17 | Report other analyses done — e.g., analyses of subgroups and interactions, and sensitivity analyses | Not applicable |
| **Discussion** |  |  |  |
| **Key results** | 18 | Summarise key results with reference to study objectives | Discussion, paragraph 1 |
| **Limitations** | 19 | Discuss limitations of the study, taking into account sources of potential bias or imprecision. Discuss both direction and magnitude of any potential bias | Discussion, last paragraph (single-center, small sample, individualized strategies, no serial AQP4-IgG titers) |
| **Interpretation** | 20 | Give a cautious overall interpretation of results considering objectives, limitations, multiplicity of analyses, results from similar studies, and other relevant evidence | Discussion, paragraphs 1–5 and Conclusion (hypothesis-generating, not confirmatory) |
| **Generalisability** | 21 | Discuss the generalisability (external validity) of the study results | Discussion, limitations paragraph (single-center Japanese cohort; findings may not generalize to other populations) |
| **Other information** |  |  |  |
| **Funding** | 22 | Give the source of funding and the role of the funders for the present study and, if applicable, for the original study on which the present article is based | Declarations, Funding (AMED, MHLW, JSPS KAKENHI) |
